# Lawrence B. Slobodkin (1928–2009): Integrating Theory, Models, and Experiments in Ecology

**DOI:** 10.1371/journal.pbio.1000261

**Published:** 2009-12-22

**Authors:** Douglas J. Futuyma, Robert K. Colwell

**Affiliations:** 1 Department of Ecology and Evolution, Stony Brook University, Stony Brook, New York, United States of America; 2 Department of Ecology and Evolutionary Biology, University of Connecticut, Storrs, Connecticut, United States of America

Lawrence B. Slobodkin, a key figure in the development of the modern science of ecology, passed away on September 12, 2009, at age 81. His innovative thinking and research, provocative teaching, and visionary leadership helped transform ecology into a modern science, with deep links to evolution.[Fig pbio-1000261-g001]

**Figure pbio-1000261-g001:**
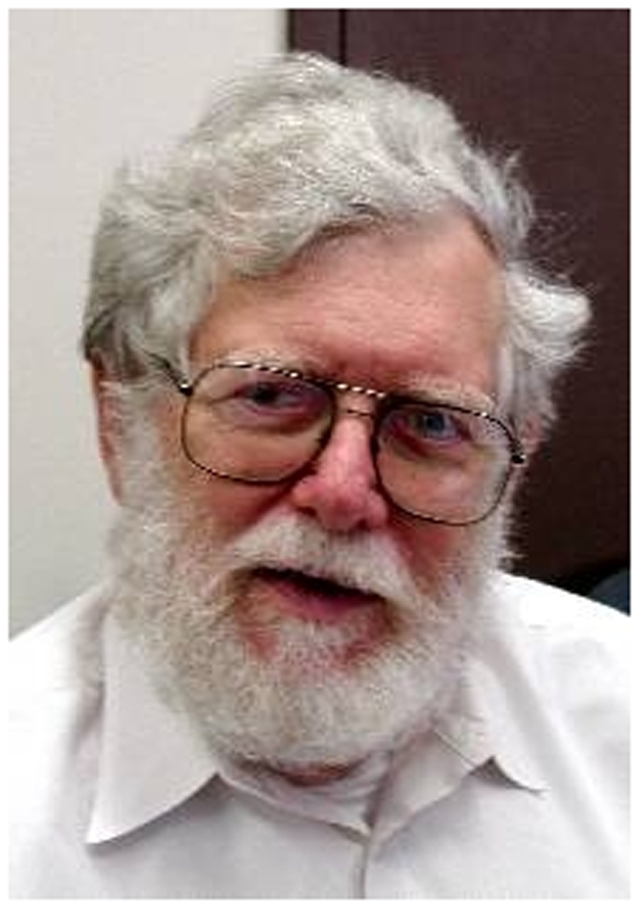
Lawrence B. Slobodkin.

A child of the Bronx, New York City, Slobodkin was strongly influenced by the artistic, intellectual, cultural, and political milieu in which he developed; his mother was a writer and his father a noted sculptor who later became a well-known illustrator and writer of children's books, including biographies of the legendary revolutionaries Garibaldi and Lenin. While absorbing the lessons of art and literature, Slobodkin developed a guiding interest in biology, which he pursued first at Bethany College in West Virginia, and later under G. E. Hutchinson at Yale, where he received his doctorate in 1951 at the age of 23. Hutchinson, one of the most renowned ecologists of the 20th century, sought broad theoretical principles for ecology, and with his students helped to build a modern theoretical and mathematical framework on foundations that Volterra and Gause had already laid. Slobodkin played an important role in developing this framework via his research, teaching, and his very influential book, *Growth and Regulation of Animal Populations* [1], which served as a blueprint for generations of students of ecology at all levels. His doctoral research, a detailed study of the role of age structure in the growth of experimental populations of the microcrustacean *Daphnia*, epitomized his approach—a quantitative experimental test of a mathematical theory that was intended to apply broadly.

After completing his Ph.D., Slobodkin worked for two years for the United States Fish and Wildlife Service, where he developed a novel, theoretically informed hypothesis for the origin of red tides. He then joined the faculty of the University of Michigan in the Department of Zoology in 1953, where he pioneered the use of calorimetry as a tool for studying the “efficiency” of energy flow in ecosystems, a field in which his groundbreaking experimental work left a permanent legacy. He initiated a research program on brown and green hydra that explored such problems as the joint role of food and predation on limiting population growth, and the continuum of species interactions that lie between mutualism and parasitism. Together with Nelson G. Hairston, Sr. and Frederick Smith, he wrote one of the most influential papers in the history of ecology, a four-page essay in *The American Naturalist* [2] that is still required reading for many students in this field. Submitted under the title “Étude” (unacceptable to the editors), HSS (as the paper is often referred to, for Hairston, Smith, and Slobodkin) offered a simple but closely reasoned hypothesis for the regulation of populations at each trophic level in terrestrial ecosystems. The “world is green,” they reasoned, despite the insatiable appetite and enormous diversity of herbivores, because herbivore populations are held in check by their own natural enemies—predators, parasitoids, parasites, and pathogens. This hypothesis was both controversial and inspiring, and stimulated much later research on tri-trophic interactions, food web dynamics, and trophic cascades. 

Larry Slobodkin's quick and sophisticated wit, infusing both his conversation and teaching, was legendary. As a graduate student teaching assistant in zoology at the University of Michigan, one of us listened to his lectures, held in a basement-level auditorium where the podium was flanked by a door to the building's loading dock. As he described the musical genius that blessed successive generations of the Bach family to illustrate principles of heredity, a great clattering of garbage cans issued from the loading area. The noise had hardly stopped when Slobodkin quipped, “the janitors here prefer Tchaikovsky.” 

By the time he moved to the State University of New York at Stony Brook in 1968, Slobodkin was one of the most distinguished ecologists in the world. The department he established there—the Department of Ecology and Evolution—was one of the first of its kind, and soon became recognized as a preeminent department in its field under his leadership. While at Stony Brook, Slobodkin served as department chair for five years and directed its graduate program for seven years, in addition to serving as co-editor of *The American Naturalist*, and writing two more books, most recently *A Citizen's Guide to Ecology* [3]. Many of the Ph.D. students he mentored first at the University of Michigan and later at Stony Brook went on to become well known ecologists, environmental scientists, and evolutionary biologists.

Among his many other activities, Slobodkin held a key post as instructor and director of a marine ecology course, taught at the Marine Biological Laboratory at Woods Hole for many years in the 1960s, that served as a training ground for prominent ecologists. He was a visiting scholar at Hebrew University, Tel Aviv University, and Ben-Gurion University, as well as the Weizman Institute, in Israel, twice a Guggenheim Fellow, twice a Fulbright Fellow, and a fellow of the Woodrow Wilson International Center for Scholars. He was honored by being elected as Fellow of the American Academy of Arts and Sciences and as Foreign Member of the Linnaean Society of London. He was president of the American Society of Naturalists in 1985 and the Society for General Systems Research in 1969. 

In 2005, Slobodkin, then Emeritus Professor of Ecology and Evolution at Stony Brook University, was named Eminent Ecologist by the Ecological Society of America. When asked to write a piece for the Ecological Society of America's series, *What Do Ecologists Do?*, after receiving the award, Slobodkin wrote, “My own advice on career development is that there are three career paths open and it is wise to excel at one of them: the first is to become an expert on some group of organisms that excites you…. Second, you [could] become very good at the most popular current techniques at the highest technical level you can imagine. In contrast, you can take the third, and most dangerous, path. You can strenuously avoid doing what everyone else is doing and search for new ideas and new tests for old ideas.” Larry Slobodkin followed, with intensity, that third and most perilous path.

Slobodkin's research accomplishments were broad. He was an innovative thinker whose ideas provided the foundations for many topics that are still studied today. His research and writings were infused with erudition and wit that extended to his lectures and conversations. No one who knew him will forget his ability to express an idea, explanation, or his own experiences in the most incisive and humorous way. His ability to recall poetry, biblical references, arcane historical anecdotes, or Jewish jokes to fit any situation was legendary. He was vocally liberal and sensitive to the needs and feelings of immigrants and others who he thought might feel marginalized. He was an inspiration to students, colleagues, friends, and family.
